# Severe Vision Impairment and Its Association with COVID-19 Susceptibility, Severity, and Mortality: A Population-Based Cohort Study

**DOI:** 10.3390/jcm15176563

**Published:** 2026-08-25

**Authors:** Young Kook Kim, Su Hwan Kim, June Ho Lee, Chae Young Oh, Hajoung Lee, Goneui Kang, Sooyeon Choe, Jieun Cheon, Hyung-Jin Yoon, Ahnul Ha

**Affiliations:** 1 Department of Ophthalmology, Seoul National University Hospital, Seoul 03080, Republic of Korea; 2 Department of Preventive Medicine, College of Medicine, The Catholic University of Korea, Seoul 06591, Republic of Korea; 3 Seoul National University College of Medicine, Seoul National University, Seoul 03080, Republic of Korea; 4 Department of Statistics, Sungkyunkwan University, Seoul 03063, Republic of Korea; 5 Department of Statistics, Seoul National University, Seoul 08826, Republic of Korea; 6 Department of Ophthalmology, Ajou University Hospital, Ajou University School of Medicine, Suwon 16499, Republic of Korea; 7 Sociocultural Research Division, Jeonbuk Institute, Jeonju 55068, Republic of Korea; 8 Medical Bigdata Research Center, Seoul National University College of Medicine, Seoul 03080, Republic of Korea; 9 Department of Ophthalmology, Jeju National University College of Medicine, Jeju-si 63243, Republic of Korea

**Keywords:** COVID-19, infection, visual impairment, blindness

## Abstract

**Background/Objectives:** We investigated the associations of severe vision impairment (SVI) with SARS-CoV-2 infection and COVID-19 outcomes using the nationwide K-COV-N database, which links Korea Disease Control and Prevention Agency SARS-CoV-2 testing records with National Health Insurance Service data. **Methods:** Adults aged ≥20 years with laboratory-confirmed SARS-CoV-2 infection and matched controls who either tested negative or were untested but had no documented SARS-CoV-2 infection between 8 October 2020, and 31 December 2021, were included. Pre-existing SVI was defined using an inclusive algorithm based on either a best-corrected visual acuity <6/60 in the better-seeing eye in the health-checkup database or legal SVI recorded in the National Disability Registration System before the COVID-19 observation period. Propensity score matching (1:30) was used to evaluate SARS-CoV-2 test positivity in the overall cohort and severe COVID-19 outcomes among infected individuals. **Results:** The study included 560,182 SARS-CoV-2-positive individuals and 1,677,364 matched controls. After adjustment, SVI was associated with a trend toward higher SARS-CoV-2 test positivity (adjusted odds ratio [aOR], 1.09; 95% confidence interval [CI], 0.99–1.20). Among infected individuals, SVI was associated with increased odds of severe COVID-19 illness (aOR, 3.05; 95% CI, 2.59–3.58) and COVID-19-related death (aOR, 1.39; 95% CI, 1.13–1.71), with stronger associations during the Delta variant period. **Conclusions:** These findings suggest that SVI is associated with poorer COVID-19 outcomes and may increase susceptibility to SARS-CoV-2 infection.

## 1. Introduction

During the peak of the COVID-19 pandemic, caused by SARS-CoV-2, it had far-reaching consequences for global health systems [1]. Although COVID-19 often causes mild symptoms that are typical of other respiratory infections, it has also demonstrated the capacity to induce severe symptoms that can lead to fatal outcomes in certain patient populations [2]. Baseline characteristics, including old age [3], male sex [4], and obesity [5], as well as underlying comorbidities such as cardiovascular disease [6,7], respiratory disease [6,7] and cancer [8,9], have demonstrated an association with increased susceptibility to COVID-19 and COVID-19-associated death. Notably, accumulating research has identified multiple risk factors for COVID-19, reflecting complex interactions among the pathogen, the host, and the environment [10].

The global growth and aging of populations have resulted in a rising prevalence of individuals affected by visual impairment (VI) [11]. The World Health Organization (WHO) has emphasized the need for integrated, people-centered eye care to address the growing global burden of VI [12]. Individuals with severe vision impairment (SVI) represent a particularly vulnerable population because of substantial limitations in mobility, communication, access to health information, and healthcare utilization. In relation to health outcomes, SVI has implications that extend beyond the confines of clinical ophthalmology [13]. Longitudinal studies have shown that VI is a risk factor associated with cognitive decline [14], reduced physical activity [15], depression [16,17], social isolation [18], and suicide mortality [19].

Individuals with SVI faced heightened challenges during the COVID-19 pandemic, rendering them a vulnerable group [20,21]. Previous studies suggest that they exhibit higher rates of medical conditions [22], potentially increasing their susceptibility to COVID-19. Moreover, research indicates that individuals with VI may engage less in positive health behaviors [23,24], which may also be true for health behaviors relevant to COVID-19 prevention. They also encounter greater obstacles in accessing and implementing preventive measures against COVID-19 [25]. Relying on tactile information for environmental perception, moreover, further exacerbates their risk of contracting the virus [20]. In addition, public health information regarding COVID-19 was frequently delivered through written materials, signage, and digital visual content, which may not have been readily accessible to individuals with SVI. These factors collectively suggest that individuals with SVI may be at increased risk of both SARS-CoV-2 infection and adverse COVID-19 outcomes.

However, despite these plausible mechanisms, population-based evidence regarding the association between SVI and COVID-19 outcomes remains limited. To address this knowledge gap, we conducted a nationwide population-based cohort study in South Korea to investigate whether pre-existing SVI is associated with susceptibility to SARS-CoV-2 infection and severe COVID-19 outcomes, including mortality.

## 2. Methods

### 2.1. Study Design, Setting, and Data Sources

We conducted a population-based cohort study utilizing a K-COV-N (Korea Disease Control and Prevention Agency [KDCA]-COVID19-National Health Insurance Service [NHIS]) cohort. During the pandemic, COVID-19-related medical care in South Korea was provided under a government-mandated system that covered all confirmed patients nationwide. The above-noted K-COV-N cohort was specifically constructed using the SARS-CoV-2 test-positive patient registry of the KDCA and controls from the NHIS. This cohort integrates COVID-19-related information with comprehensive longitudinal healthcare data to facilitate research on the prevention and management of COVID-19. The dataset comprises: (1) demographic characteristics; outpatient and inpatient healthcare utilization records—including healthcare facility visits, diagnostic codes, procedures, and prescription information—as well as pharmacy claims and lifestyle-related variables, as collected by NHIS between 1 January 2009, and 31 December 2021; and (2) COVID-19-specific data provided by the KDCA and the Ministry of Health and Welfare, including vaccination details (dates, doses, and vaccine types) and confirmed SARS-CoV-2 infection outcomes such as diagnosis, mortality, and infection routes, available through 31 December 2021.

This study was approved by the Institutional Review Board of Seoul National University Hospital in Seoul, South Korea (E-2311-078-1484), and was conducted in accordance with the principles of the Declaration of Helsinki. This study used de-identified data from the NHIS database, which is accessible to researchers upon approval of a research proposal and compliance with NHIS data access requirements. This study was reported in accordance with the Strengthening the Reporting of Observational Studies in Epidemiology (STROBE) guidelines.

### 2.2. Study Population

This analysis encompassed all South Korean adults aged 20 years or older with laboratory-confirmed SARS-CoV-2 infection between 8 October 2020 and 31 December 2021, as documented in the K-COV-N database. To form the control group, three control individuals (including both untested individuals with no documented SARS-CoV-2 infection and test-negative individuals) of the same age and sex were randomly selected and matched with each SARS-CoV-2-positive individual. SARS-CoV-2 infection was laboratory confirmed by real-time PCR testing of nasal/pharyngeal swabs following KDCA-approved protocols and WHO guidance.

### 2.3. Exposure

Coexisting SVI was ascertained through two distinct data sources linked to the K-COV-N database. First, SVI was identified when the best-corrected visual acuity (BCVA) of the better-seeing eye was <6/60 in the health checkup database. Second, SVI was identified through the National Disability Registry, which records individuals meeting the legal criteria for SVI under South Korean regulations. Under these regulations, legal SVI is defined as meeting at least one of the following criteria that has persisted for six months or longer despite treatment and is not reversible with medical or surgical interventions, with the exception of keratoplasty: (1) BCVA ≤ 6/100 in the better eye, or (2) a visual field constricted to ≤5 degrees from the visual axis in both eyes. Registration in the National Disability Registry requires submission of a medical certificate issued by an ophthalmologist documenting BCVA, visual field findings, and the underlying causes of SVI.

For the primary analysis, the two ascertainment sources were combined using an inclusive algorithm: fulfillment of either criterion before 8 October 2020 was sufficient for classification as SVI, and no hierarchy was applied between the two sources. These groups were combined because both experience severe, persistent functional limitations that may similarly affect access to healthcare and increase vulnerability during the COVID-19 pandemic. To ensure that SVI preceded the outcomes, only SVI documented before the COVID-19 outcome assessment period (8 October 2020–31 December 2021) was classified as the exposure. COVID-19 outcomes were then evaluated according to pre-existing SVI status.

### 2.4. Covariates

Data on patient demographics, including age, sex, area of residence, income level, and number of COVID-19 vaccinations, were collected from insurance eligibility records. Rural or urban residence was determined as previously reported [26]. The presence of comorbidities such as hypertension, diabetes, cardiovascular disease, cerebrovascular disease, chronic obstructive pulmonary disease (COPD), asthma, and chronic kidney disease was ascertained by requiring at least two healthcare claims recorded within a one-year interval using International Classification of Diseases and Related Health Problems, Tenth Revision (ICD-10) codes (Table S1). The Charlson comorbidity index score was calculated based on the ICD-10 codes. The Charlson comorbidity index was included to capture a broad range of overall comorbidity burden, including conditions not represented by the individually modeled indicators, whereas the individual comorbidity variables were retained to account directly for specific conditions strongly associated with COVID-19 outcomes. Age, sex, area of residence, income level, number of COVID-19 vaccinations, and the other covariates included in the propensity score and outcome models had no missing values (0% missing). Therefore, no imputation or other missing-data procedure was required, and no participant was excluded because of missing covariate data.

We also obtained information on tertiary planning unit (TPU)-level social capital factors [27], measured through trust (towards community and government policy), norms (reciprocity and moral norms), and networks (number of religious organizations per capita and nonprofit organizations per 10,000 people), as obtained from the Korean Statistical Information Service.

### 2.5. Statistical Analyses

To minimize potential confounding and achieve balance in baseline characteristics between the two groups, we employed propensity score matching (PSM) to compare SARS-CoV-2 test positivity and critical clinical outcomes of COVID-19. Because SVI was rare in the source cohort (3805 of 2,237,546 participants; 0.17%), greedy nearest-neighbor matching permitted up to 30 participants without SVI per participant with SVI to make use of the large control pool and improve precision while retaining exposed participants. Matching was conducted separately in the entire cohort (*n* = 2,237,546) and the SARS-CoV-2-positive subgroup (*n* = 560,182). The propensity score model was used to balance measured baseline covariates rather than to predict SVI status. Matching adequacy was therefore assessed using the propensity score distributions and post-matching standardized mean differences (SMD), with an absolute SMD < 0.1 considered indicative of acceptable balance.

In the main analysis, SVI was the exposure; the primary outcome was SARS-CoV-2 test positivity among all individuals in Cohort 1. In Cohort 2, which included only individuals with confirmed SARS-CoV-2 infection, secondary outcomes encompassed severe COVID-19-related clinical events—including admission to the intensive care unit, need for invasive mechanical ventilation, and COVID-19-related death (ascertained through 31 March 2022). Logistic regression models were used to assess the odds of primary and secondary outcomes between the groups with and without SVI, with adjusted odds ratios (aORs) and 95% confidence intervals (CIs) estimated in each matched cohort. The adjusted covariates were age, sex, area of residence, income level, number of COVID-19 vaccinations, TPU-level social capital factors, Charlson comorbidity index, diabetes, cardiovascular disease, cerebrovascular disease, COPD, asthma, hypertension, and chronic kidney disease.

Additional analyses were performed to compare risk according to the prevalence of the dominant strain of SARS-CoV-2 (the less prevalent period of the Delta strain [% Delta strain < 50%; 8 October 2020–25 July 2021] versus the highly prevalent period of the Delta strain [% Delta strain ≥ 50%; 26 July 2021–31 December 2021]) [28]. Statistical analyses were carried out with SAS (version 9.4) and R statistical software (version 3.1.1), with statistical significance defined by a two-sided *p*-value of less than 0.05.

## 3. Results

### 3.1. Demographic and Clinical Characteristics

**Figure 1:**
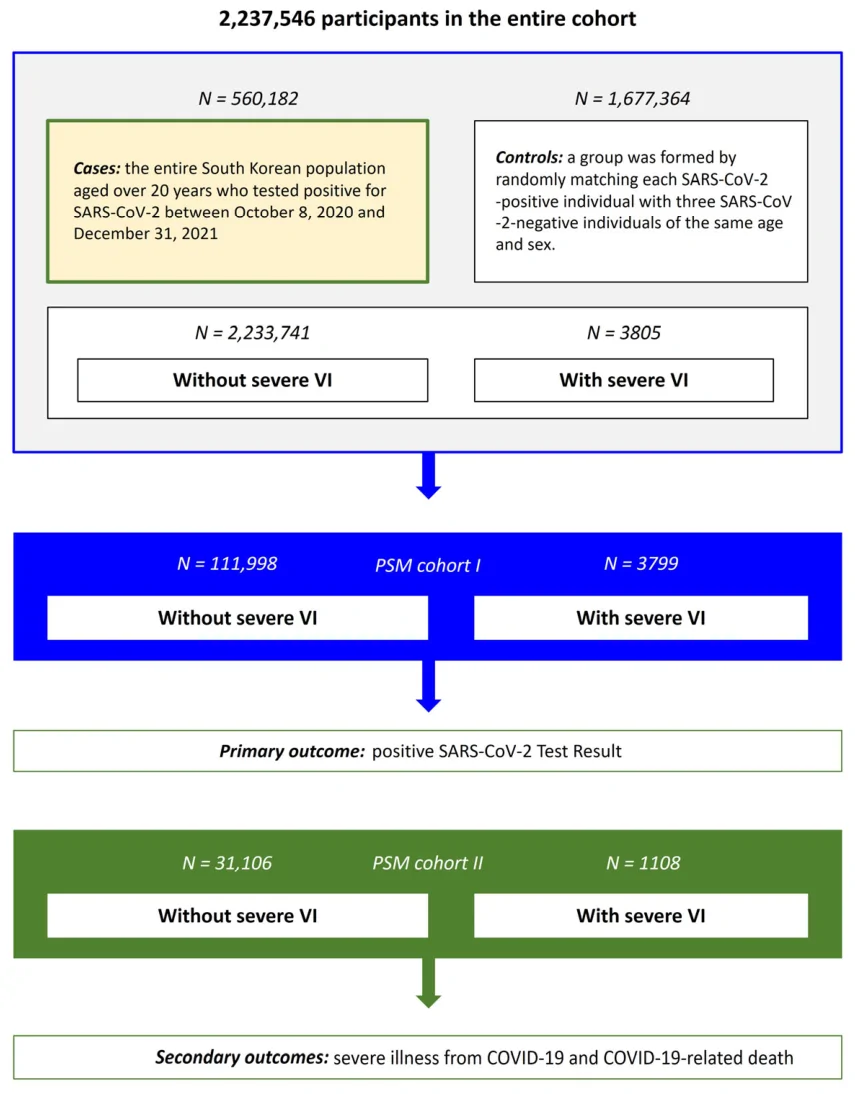
Flow diagram of participant selection. We conducted a population-based cohort study utilizing the K-COV-N (Korea Disease Control and Prevention Agency-COVID-19-National Health Insurance Service) cohort, which includes all South Korean residents aged over 20 years who tested positive for SARS-CoV-2 between 8 October 2020 and 31 December 2021. PSM = propensity score matching; VI = visual impairment.

A total of 560,182 SARS-CoV-2-positive patients (mean [SD] age, 41.9 [21.6] years; 290,920 men [52.0%]) and 1,677,364 matched individuals who were negative for SARS-CoV-2 were included. Among the total cohort, 2,233,741 individuals (99.8%) did not have SVI, with a mean age of 41.0 (SD: 21.6) years; 1,150,279 (51.5%) were male. On the other hand, 3805 participants (0.2%) had SVI, with a mean age of 65.2 (SD: 17.8) years; 1868 (49.1%) were male (Table 1 and Figure 1). Patients with SVI, compared with those without, exhibited higher proportions of residing in rural areas (42.5% vs. 34.7%), having a low-income status (38.0% vs. 16.5%), receiving the COVID-19 vaccine more than three times (56.3% vs. 45.1%), and having a history of diabetes, hypertension, cardiovascular disease, cerebrovascular disease or chronic kidney disease. Among the individuals without SVI (*n* = 2,233,741), 25.0% (*n* = 559,069) tested positive for SARS-CoV-2. By contrast, among those with SVI (*n* = 3805), 29.3% (*n* = 1113) were identified as SARS-CoV-2-positive (Table 1).

### 3.2. COVID-19 Morbidity and Its Association with SVI

**Figure 2:**
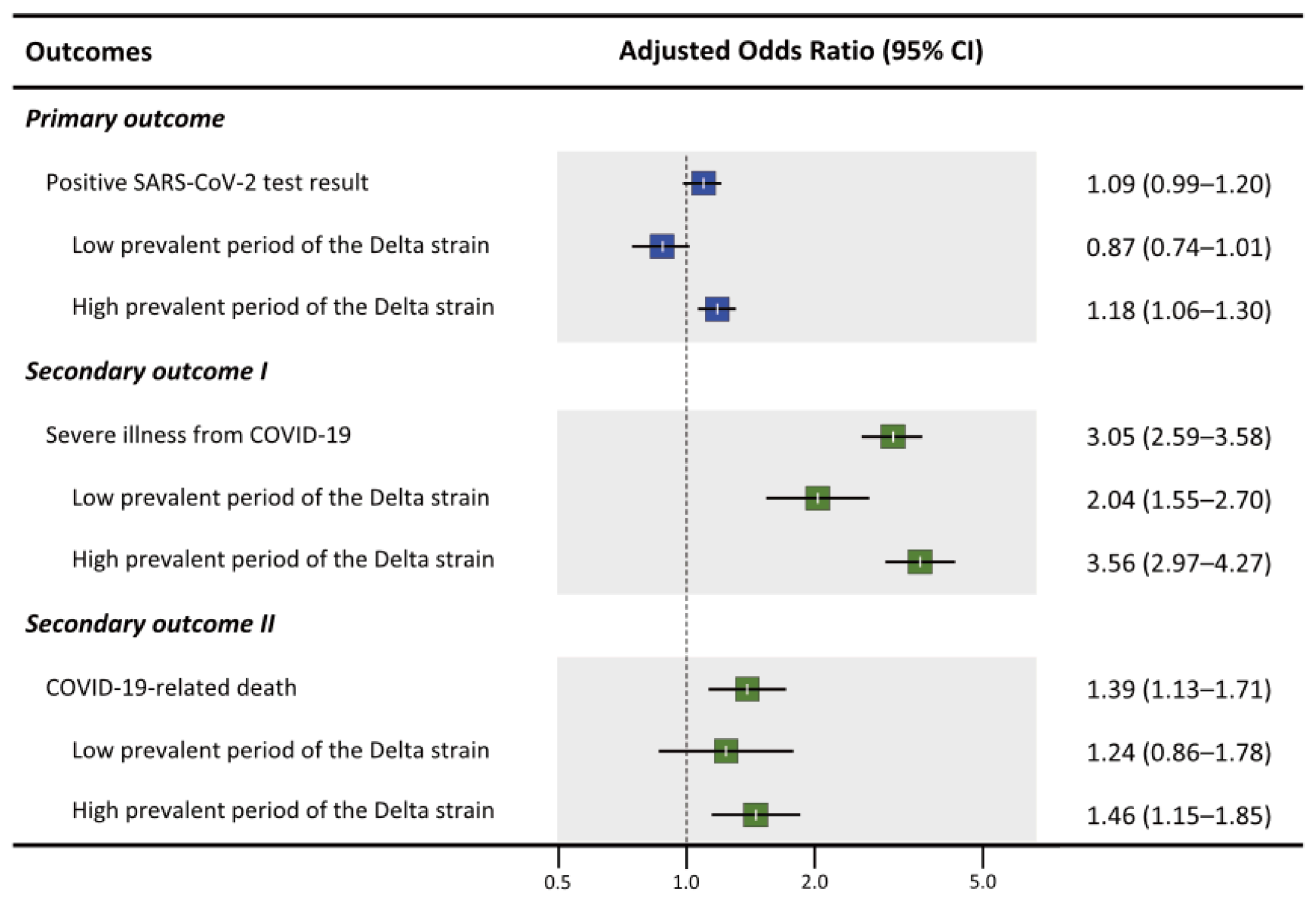
Association of severe VI with positive SARS-CoV-2 test results in entire cohort (*n* = 2,237,546), association of severe VI with COVID-19 clinical outcomes among patients who had tested positive (*n* = 560,182), and subgroup analysis comparing risk based on prevalence of dominant strain of SARS-CoV-2. CI = confidence interval.

The overall matched cohort included 3799 participants with SVI and 111,998 participants without SVI (total *n* = 115,797) and is described in Table S2. Baseline characteristics showed adequate balance between groups after matching, with all SMDs being less than 0.1. The positivity rate of SARS-CoV-2 testing was 27.0% (30,214 patients) in individuals without SVI and 29.2% (1109 patients) in those with SVI. Following adjustment for confounders, the aOR for SARS-CoV-2 test positivity among participants with SVI was 1.09 (95% CI, 0.99–1.20; Figure 2).

### 3.3. COVID-19 Severity and Mortality and Their Associations with SVI

Among the total cohort of 560,182 individuals who had tested positive for SARS-CoV-2, 99.8% (559,069 individuals) did not have SVI, while 0.2% (1113 participants) had SVI (Table S3). Within the COVID-19 patient population, 4.3% (23,813 patients) without SVI and 26.6% (296 patients) with SVI experienced severe illness attributed to COVID-19. Furthermore, 1.7% (9381 patients) without SVI and 11.1% (123 patients) with SVI suffered COVID-19-related death.

Among SARS-CoV-2-positive participants, the matched cohort included 1108 participants with SVI and 31,106 participants without SVI. Baseline characteristics were comparable between the matched groups, all SMDs being less than 0.1. The propensity score distributions are presented in Figure S1, and the post-matching SMDs are reported in Table 2 and Table S2.

The incidence of severe illness attributed to COVID-19 was 12.6% (3927 patients) in individuals without SVI and 26.4% (293 patients) in those with SVI (Table 2). After multivariable adjustment, the aOR for severe illness from COVID-19 in patients with SVI was 3.05 (95% CI, 2.59–3.58) (Figure 2). The incidence of COVID-19-related death was 8.0% (2493 patients) in individuals without SVI and 10.9% (121 patients) in those with SVI (Table 2). SVI was associated with a higher likelihood of COVID-19–related death, with an aOR of 1.39 (95% CI, 1.13–1.71, Figure 2).

### 3.4. Subgroup Analyses Based on Viral Strain

We performed subgroup analyses to assess the risk of SARS-CoV-2 infection based on the prevalence of the dominant viral strain. Our findings revealed that among patients with SVI, the aOR for positive SARS-CoV-2 test results was significantly greater during periods of high Delta strain prevalence than during periods of low Delta strain prevalence (aOR 1.18, 95% CI 1.06–1.30 vs. 0.87, 95% CI 0.74–1.01; Figure 2). Moreover, the risk of experiencing severe illness from COVID-19 was significantly greater among individuals with SVI during the period of high Delta strain prevalence than during the period of low Delta strain prevalence (aOR 3.56 [95% CI 2.97–4.27] vs. 2.04 [95% CI 1.55–2.70]; Figure 2). COVID-19-associated death was also greater among individuals with SVI during the period of high Delta strain prevalence than during the period of low Delta strain prevalence (aOR 1.46 [95% CI 1.15–1.85] vs. 1.24 [95% CI 0.86–1.78]; Figure 2).

We analyzed the moderation effect of the Delta strain on the association between SVI and both severe illness and mortality among COVID-19-positive cases. The results indicated a significant interaction between the Delta strain and SVI on COVID-19 severe illness (*p* = 0.017), but not on mortality (*p* = 0.934, Table S4).

### 3.5. Sensitivity Analyses

Within the overall study population, analyses using a 1:30 case–control PSM cohort consistently demonstrated an association between SVI and worse COVID-19-related clinical outcomes (*n* = 2,237,546, Table S5). Furthermore, consistent results were observed when the primary and secondary outcomes were evaluated using PSM with different case–control ratios (1:1, 1:3, 1:5, and 1:10; Table S6).

## 4. Discussion

Our findings revealed no significant overall association between SVI and COVID-19 infection, although a heightened risk was observed during the Delta-dominant period in the South Korean population. Moreover, we identified a significant association between SVI and adverse clinical outcomes, including mortality. Notably, individuals with SVI had higher vaccination coverage than those without SVI (56.3% vs. 45.1% for ≥3 doses). The persistence of an increased risk of adverse clinical outcomes despite this higher vaccination coverage may further support the association between SVI and COVID-19-related vulnerability.

The higher risk of COVID-19 associated with SVI appears to stem from the unique challenges and impacts of the pandemic [29]. One factor that could have contributed to this risk is the inherent challenge of practicing social distancing [30]. Owing to their reliance on vision for maintaining distance, individuals with SVI may have encountered difficulties adhering to the relevant regulations. Also, impairment of vision makes one rely on other senses, especially touch. According to a survey conducted in Canada in May 2020, 68% of people with vision loss agreed that they were concerned by the inevitable incidental contact experienced in public [31]. Touching mobility canes, reading braille, and pressing a button for an acoustic signal to cross a street may all lead to higher danger of COVID-19 infection.

Another burden for the visually impaired in the COVID-19 pandemic is the information gap and difficulty accessing healthcare [32]. Nowadays, a considerable amount of information is delivered through the internet and social networking, but many websites rely on visual content for sufficient information. In addition, important public health information is often communicated through written notices, signage, printed materials, and digital visual displays that may not be readily accessible to individuals with SVI. Without accessible formats such as large-print, braille, audio, or screen-reader–compatible materials, obtaining crucial information regarding COVID-19—including updated policies, preventive measures, testing procedures, and appropriate responses following infection—can be challenging, hindering their ability to comply with guidelines and prepare emergency plans [20].

Notably, our findings demonstrated that individuals with SVI had an elevated susceptibility to SARS-CoV-2 infection and were at increased risk of unfavorable clinical outcomes, particularly during periods dominated by the Delta variant. The Delta variant has exhibited increased transmissibility compared with previous variants, even among fully vaccinated individuals, with estimates suggesting that it is approximately twice as contagious as earlier strains [33]. Moreover, prior studies investigating the inherent severity of various SARS-CoV-2 variants have indicated higher levels of disease severity associated with the Delta strain [34]. The risk of hospitalization was found to be between 1.85 and 3.0 times higher for Delta than the Alpha variant [35,36,37]. Based on this information, it can be inferred that the more potent the virus, the greater the impact on vulnerable groups, including those with SVI.

Participants with SVI were older and had higher crude prevalences of diabetes, hypertension, cardiovascular and cerebrovascular disease, and chronic kidney disease, all of which may increase the risk of adverse COVID-19 outcomes. Propensity score matching was performed to balance these measured baseline differences, and these factors were also included in the adjusted outcome models. After matching, all reported SMDs, including those for age and vascular and renal comorbidities, were below 0.1. Nevertheless, residual confounding may remain because claims data did not fully capture disease duration, severity, clinical control, frailty, or functional status, which may influence both SVI and the risk of SARS-CoV-2 infection.

There are several notable study limitations. First, SVI was ascertained using two sources with different eligibility criteria, and diagnoses could not be confirmed by individual chart review. Source-specific concordance and the robustness of the findings to a registry-only SVI definition could not be evaluated. Individuals with the most severe visual impairment may also have been less likely to participate in routine health examinations, resulting in underascertainment that may not have been fully addressed by linkage to the disability registry. Exposure misclassification may therefore have resulted in either underestimation or overestimation of the associations. Second, it is possible that the control group included individuals who were not tested for SARS-CoV-2 and were presumed to be uninfected due to the lack of documented symptoms. During the study period, in South Korea, vaccination, SARS-CoV-2 testing, and treatment for positive cases were provided free of charge to all citizens [38]. As a result, we believe that the number of COVID-19-infected patients in the control group is not significant, and that this does not alter the main message of our study. Third, despite adjustment for multiple covariates and the use of PSM, the possibility of residual confounding due to unmeasured variables, including comorbidities, medication exposure, and lifestyle factors, remains. Although the Charlson comorbidity index partially overlaps with several individual comorbidity indicators, the two representations capture different aspects of comorbidity. The overlap may have reduced precision through collinearity, but it would not be expected to bias the estimated association with SVI systematically in a specific direction. Future studies should explore clinical factors that could affect COVID-19 susceptibility in visually impaired populations. Fourth, detailed symptom data and viral load information were unavailable due to the use of claims-based data. Additional research is needed to elucidate the associations between SVI and various symptoms and signs related to COVID-19, as well as viral load. Fifth, our findings pertain specifically to the South Korean population; thus, the generalizability to other populations remains uncertain. According to recent systematic reviews, ethnicity may play a role in COVID-19 infection and its clinical outcomes [39]. Lastly, although our study found associations between SVI and COVID-19 outcomes, it was unable to establish a causal relationship due to its observational nature.

Despite these limitations, our results offer some new suggestions to public health policy. Those with SVI should be considered to be a high-risk group for COVID-19, along with those of old age or with underlying comorbidities. In particular, special attention is required after testing positive for SARS-CoV-2, as the risk for severe illness and death seems to be remarkably high in this population. In addition, the associations between SVI and COVID-19 susceptibility, severity, and morbidity imply possible associations between SVI and other infectious diseases. Despite the Independent Panel for Pandemic Preparedness and Response (convened by the WHO)’s recommendations for ending the pandemic and preventing future ones [40], another pandemic may strike. Considering the significant association between SVI and worse clinical outcomes of COVID-19, not to mention the predicted significant increase in the total VI population, careful attention paid to the VI community is needed in advance.

## 5. Conclusions

Findings from this nationwide cohort indicate that SVI was associated with an increased risk of COVID-19-associated mortality, but not with SARS-CoV-2 positivity overall. These findings highlight the need for accessible health communication and healthcare delivery strategies for individuals with SVI during infectious disease outbreaks and other public health emergencies. In addition, further studies are warranted to confirm these findings and to investigate the biological and clinical mechanisms connecting SVI with susceptibility to infectious diseases.:

## Figures and Tables

**Table 1:** Demographic and clinical characteristics of patients without and with severe visual impairment in the entire cohort.

Characteristics	Korean Nationwide Cohort (*n* = 2,237,546)		
	Overall	Without Severe VI	With Severe VI
Total, *n* (%)	2,237,546 (100.0)	2,233,741 (99.8)	3805 (0.2)
Age, years (SD)	41.0 (21.6)	41.0 (21.6)	65.2 (17.8)
20–39, *n* (%)	1,480,276 (66.2)	1,478,544 (66.2)	1732 (45.5)
40–59, *n* (%)	582,781 (26.0)	581,814 (26.0)	967 (25.4)
≥60, *n* (%)	174,489 (7.8)	173,383 (7.8)	1106 (29.1)
Sex, *n* (%)			
Male	1,152,147 (51.5)	1,150,279 (51.5)	1868 (49.1)
Female	1,085,399 (48.5)	1,083,462 (48.5)	1937 (50.9)
Region of residence, *n* (%)			
Rural	776,397 (34.7)	774,780 (34.7)	1617 (42.5)
Urban	1,461,149 (65.3)	1,458,961 (65.3)	2188 (57.5)
Low income, *n* (%)	370,291 (16.5)	368,844 (16.5)	1447 (38.0)
COVID-19 vaccination, *n* (%)			
0	610,889 (27.3)	610,084 (27.3)	805 (21.2)
1	70,378 (3.1)	70,284 (3.2)	94 (2.7)
2	547,517 (24.5)	546,752 (24.5)	765 (20.1)
≥3	1,008,762 (45.1)	1,006,621 (45.1)	2141 (56.3)
TPU level social factors			
Trust	2.44 ± 0.25	2.44 ± 0.25	2.45 ± 0.26
Reciprocity norm	550.6 ± 208.5	550.6 ± 208.4	572.4 ± 253.8
Moral norms	30.6 ± 8.88	30.6 ± 8.88	30.7 ± 9.72
Number of religious organizations per capita	1.39 ± 0.67	1.39 ± 0.67	1.59 ± 0.84
Number of nonprofit organizations per 10,000 people	0.29 ± 0.30	0.29 ± 0.30	0.31 ± 0.34
Comorbidities			
Diabetes mellitus, *n* (%)	193,513 (8.6)	192,620 (8.6)	893 (23.5)
Cardiovascular disease, *n* (%)	22,578 (1.0)	22,453 (1.0)	125 (3.3)
Cerebrovascular disease, *n* (%)	8606 (0.4)	8530 (0.4)	76 (2.0)
COPD, *n* (%)	183,365 (8.2)	183,118 (8.2)	247 (6.5)
Asthma, *n* (%)	52,719 (2.4)	52,664 (2.4)	55 (1.5)
Hypertension, *n* (%)	390,959 (17.5)	389,109 (17.4)	1850 (48.6)
Chronic kidney disease, *n* (%)	66,193 (3.0)	65,582 (2.9)	611 (16.1)
Charlson comorbidity index, *n* (%)			
0	1,018,438 (45.5)	1,017,840 (45.6)	598 (15.7)
1	679,400 (30.4)	678,541 (30.4)	859 (22.6)
≥2	539,708 (24.1)	537,360 (24.0)	2348 (61.7)
Positive SARS-CoV-2 test result, *n* (%)	560,182 (25.0)	559,069 (25.0)	1113 (29.3)
Alpha strain	152,453 (6.8)	152,161 (6.8)	292 (7.7)
Delta strain	407,729 (18.2)	406,908 (18.2)	821 (21.6)

VI, vision impairment; SD, standard deviation; TPU, tertiary planning unit; COPD, chronic obstructive pulmonary disease.

**Table 2:** Demographic and clinical characteristics of SARS-CoV-2–positive participants with and without severe visual impairment after propensity score matching.

Characteristics	Without Severe VI	With Severe VI	SMD
Total, *n* (%)	31,106 (96.6)	1108 (3.4)	
Age, years (SD)	64.6 (17.07)	65.2 (17.3)	0.019
20–39, *n* (%)	12,828 (41.2)	438 (39.5)	0.052
40–59, *n* (%)	9296 (29.9)	324 (29.2)	
≥60, *n* (%)	8982 (28.9)	346 (31.2)	
Sex, *n* (%)			0.021
Male	16,363 (52.6)	571 (51.5)	
Female	14,743 (47.4)	537 (48.5)	
Region of residence, *n* (%)			0.047
Rural	10,279 (33.1)	390 (35.2)	
Urban	20,827 (67.0)	718 (64.8)	
Low income, *n* (%)	10,997 (35.4)	417 (37.6)	0.053
COVID-19 vaccination, *n* (%)			0.017
0	12,646 (40.7)	452 (40.8)	
1	1673 (5.4)	59 (5.3)	
2	14,420 (46.4)	508 (45.9)	
≥3	2367 (7.6)	89 (8.0)	
TPU level social factors			
Trust	2.44 ± 0.25	2.44 ± 0.24	0.010
Reciprocity norms	538.9 ± 226.4	539.6 ± 227.7	0.003
Moral norms	31.5 ± 11.04	31.6 ± 10.7	0.010
Number of religious organizations per capita	1.43 ± 0.74	1.47 ± 0.77	0.054
Number of nonprofit organizations per 10,000 people	0.32 ± 0.39	0.32 ± 0.40	0.001
Comorbidities			
Diabetes mellitus, *n* (%)	7325 (23.6)	276 (24.9)	0.037
Cardiovascular disease, *n* (%)	1255 (4.0)	51 (4.6)	0.035
Cerebrovascular disease, *n* (%)	510 (1.6)	18 (1.6)	0.001
COPD, *n* (%)	2078 (6.7)	72 (6.5)	0.007
Asthma, *n* (%)	403 (1.3)	14 (1.3)	0.002
Hypertension, *n* (%)	14,968 (48.1)	549 (49.6)	0.032
Chronic kidney disease, *n* (%)	4485 (14.4)	190 (17.2)	0.092
Charlson comorbidity index, *n* (%)			0.018
0	4230 (13.6)	153 (13.8)	
1	6650 (21.4)	229 (20.7)	
≥2	20,226 (65.0)	726 (65.5)	
Severe illness from COVID-19, *n* (%)	3927 (12.6)	293 (26.4)	
COVID-19-related death, *n* (%)	2493 (8.0)	121 (10.9)	

VI, vision impairment; SMD, standardized mean differences; SD, standard deviation; TPU, tertiary planning unit; COPD, chronic obstructive pulmonary disease. Propensity score-matched covariates: age; sex; region of residence; history of diabetes mellitus, cardiovascular disease, cerebrovascular disease, COPD, asthma, hypertension, chronic kidney disease, and Charlson comorbidity index.

## Data Availability

The data used in this study are not publicly available because they contain potentially sensitive information. Access to the National Health Insurance Service database requires submission of a research proposal and approval by the National Health Insurance Service. The authors are not authorized to share these data directly.

## Supplementary Materials

The following supporting information can be downloaded at: https://www.mdpi.com/article/10.3390/jcm15176563/s1. Figure S1: Propensity score distributions demonstrating the adequacy of matching, Table S1: Definitions of Covariates, Table S2: Demographic and Clinical Characteristics of Patients Without and With Severe Visual Impairment after Propensity Score Matching, Table S3: Demographic and Clinical Characteristics of Patients Without and With Severe Visual Impairment Who Tested Positive for SARS-CoV-2, Table S4: Moderation effect of the Delta strain on the association between severe visual impairment and secondary outcomes, Table S5: Association of severe visual impairment with COVID-19 clinical outcomes in the entire cohort, Table S6: Odds ratios and 95% CIs for different case–control ratios (1:k) in propensity score matching.
