# Supplementary material for: Severe Vision Impairment and Its Association with COVID-19 Susceptibility, Severity, and Mortality: A Population-Based Cohort Study
Source: J Clin Med. 2026 Aug 25;15(17):6563. doi: 10.3390/jcm15176563 (PMC13566827; doi:10.3390/jcm15176563)
Supplement: Supplementary file 1 [file jcm-15-06563-s001.zip › jcm-4465111-supplementary.pdf]

Supplementary Material

Online-Only Figure

Online supplemental Figure S1. Propensity score distributions demonstrating the adequacy of matching

Online-Only Table

Online supplemental Table S1. Definitions of Covariates

Online supplemental Table S2. Demographic and Clinical Characteristics of Patients Without and With Severe Visual Impairment after Propensity Score Matching

Online supplemental Table S3. Demographic and Clinical Characteristics of Patients Without and With Severe Visual Impairment Who Tested Positive for SARS-CoV-2

Online supplemental Table S4. Moderation effect of the Delta strain on the association between severe visual impairment and secondary outcomes

Online supplemental Table S5. Association of severe visual impairment with COVID-19 clinical outcomes in the entire cohort

Online supplemental Table S6. Odds ratios and 95% CIs for different case-control ratios (1:k) in propensity score matching

Online supplemental Figure S1. Propensity score distributions demonstrating the adequacy of matching. A, 1:30 case-control matching across the entire cohort. B, 1:30 case-control matching among individuals who tested positive for SARS-CoV-2.

A

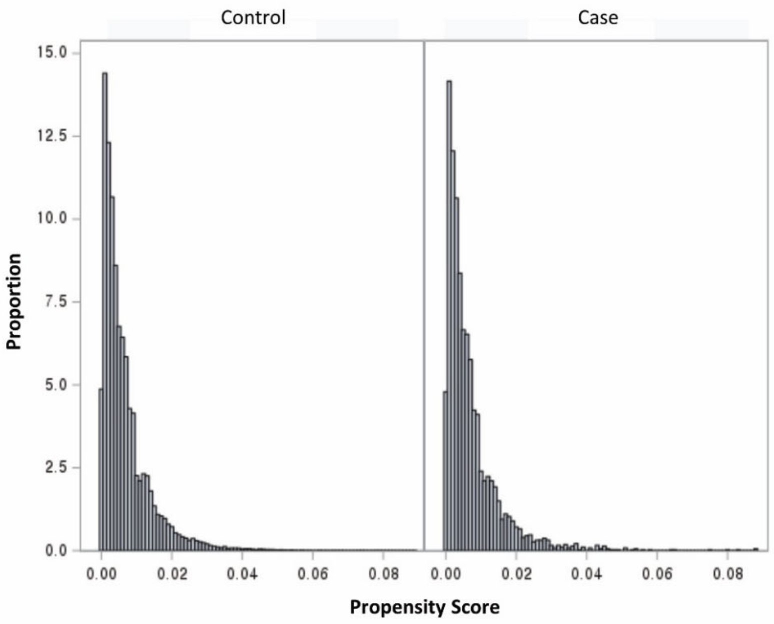

B

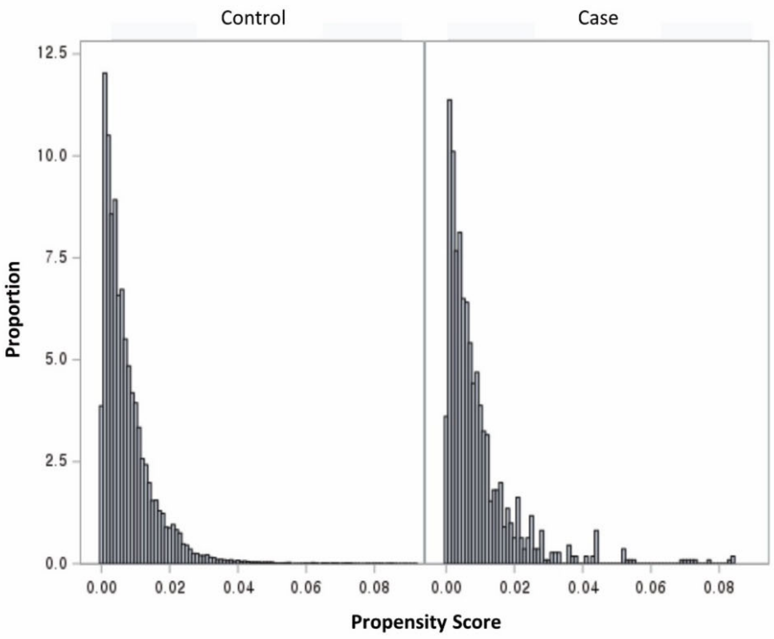

Online supplemental Table S1. Definitions of Covariates

Diagnosis	ICD-10 code and definition	Diagnostic definition
**Comorbidities**		
**Diabetes mellitus**	E11-E14; and minimum 1 prescription of anti-diabetic drugs (sulfonylureas, metformin, meglitinides, thiazolidinediones, dipeptidyl peptidase-4 inhibitors, α-glucosidase inhibitors, and insulin)	Admission≥1 or outpatient department≥2
	or fasting glucose level ≥126 mg/dL	Based on the results of the health exam
**Heart failure**	I50	Admission or outpatient department≥1
**Myocardial infarction**	I21, I22	Admission or outpatient department≥1
**Peripheral artery disease**	I70, I73	Admission or outpatient department≥2
**Cerebrovascular disease**	I60-I69	Admission or outpatient department≥2
**COPD**	J41-J44	Admission or outpatient department≥1
**Asthma**	J45, J46	Admission≥1 or outpatient department≥2
**Hypertension**	I10-I13, I15; and minimum 1 prescription of anti-hypertensive drug (thiazide, loop diuretics, aldosterone antagonist, alpha-/beta-blocker, calcium-channel blocker, angiotensin-converting enzyme inhibitor, angiotensin II receptor blocker)	Admission≥1 or outpatient department≥2
	or systolic/diastolic blood pressure ≥ 130/80 mmHg	Based on the results of the health exam
**CKD**	eGFR <60ml/min/1.73m^2	Based on the results of the health exam
	or N18.3-N18.9	
**Dyslipidemia**	E78	Admission or outpatient department≥1
	or Total cholesterol ≥ 240 mg/dL	Based on the results of the health exam
**Cancer**	C00-97 and rare intractable diseases code (V193)	Admission or outpatient department≥1
**Low income**	Income lowest 20% and medical aid	

Online supplemental Table S2. Demographic and Clinical Characteristics of Patients Without and With Severe Visual Impairment after Propensity Score Matching

Characteristics	Without Severe VI	With Severe VI	SMD
Total, n (%)	111,998 (96.7)	3,799 (3.3)	
Age, years (SD)	65.2 (17.7)	65.2 (17.8)	0.019
20-39, n (%)	51,289 (45.8)	1,732 (45.6)	
40-59, n (%)	28,657 (25.6)	964 (25.4)	0.009
≥ 60, n (%)	32,052 (28.6)	1,103 (29.0)	
Sex, n (%)			0.004
Male	55,248 (49.3)	1,867 (49.1)	
Female	56,750 (50.7)	1,932 (50.9)	
Region of residence, n (%)			0.005
Rural	47,240 (42.2)	1,611 (42.4)	
Urban	64,758 (57.8)	2,188 (57.6)	
Low income, n (%)	41,974 (37.5)	1,442 (38.0)	0.011
COVID-19 vaccination, n (%)			0.027
0	22,854 (20.4)	802 (21.1)	
1	2,707 (2.4)	94 (2.5)	
2	21,863 (19.5)	763 (20.1)	
≥ 3	64,574 (57.7)	2,140 (56.3)	
TPU level social factors			
Trust	2.45 ± 0.27	2.45 ± 0.26	0.002
Reciprocity norms	570.9 ± 247.25	572.4 ± 253.8	0.007
Moral norms	30.7 ± 9.58	30.7 ± 9.72	0.001
Number of religious organizations per capita	1.58 ± 0.83	1.59 ± 0.84	0.005
Number of nonprofit organizations per 10,000 people	0.31 ± 0.34	0.31 ± 0.34	0.001
Comorbidities			
Diabetes mellitus, n (%)	25,544 (22.8)	888(23.4)	0.016
Cardiovascular disease, n (%)	3,656 (3.3)	125 (3.3)	0.002
Cerebrovascular disease, n (%)	1,936 (1.73)	76 (2.0)	0.025
COPD, n (%)	7,025 (6.3)	247 (6.5)	0.009
Asthma, n (%)	1,570 (1.4)	55 (1.5)	0.003
Hypertension, n (%)	54,047 (48.3)	1,845 (48.6)	0.007
Chronic kidney disease, n (%)	15,831 (14.1)	605 (15.9)	0.063
Charlson comorbidity index, n (%)			0.011
0	17,312 (15.5)	598 (15.7)	
1	25,705 (23.0)	858 (22.6)	
≥ 2	68,981 (61.6)	2,343 (61.7)	
Positive SARS-CoV-2 test result, n (%)			
Alpha Strain	9,266 (8.3)	290 (7.6)	
Delta Strain	20,948 (18.7)	819 (21.6)	

VI, visual impairment; SMD, standardized mean differences; SD, standard deviation; TPU, tertiary planning unit; COPD, chronic obstructive pulmonary disease.

Online supplemental Table S3. Demographic and Clinical Characteristics of Patients Without and With Severe Visual Impairment Who Tested Positive for SARS-CoV-2

**Characteristics**	**Positive for SARS-CoV-2 (n = 560,182)**	
	**Without Severe VI**	**With Severe VI**
Total, n (%)	559,069 (99.8)	1,113 (0.2)
Age, years (SD)	41.0 (21.6)	65.3 (17.4)
20-39, n (%)	373,693 (66.8)	438 (39.3)
40-59, n (%)	143,228 (25.6)	326 (29.3)
≥ 60, n (%)	42,148 (7.6)	349 (31.4)
Sex, n (%)		
Male	287,644 (51.5)	573 (51.5)
Female	271,425 (48.6)	540 (48.5)
Region of residence, n (%)		
Rural	146,141 (26.1)	394 (35.4)
Urban	412,928 (73.9)	719 (64.6)
Low income, n (%)	94,585 (16.9)	421 (37.8)
COVID-19 vaccination, n (%)		
0	318,994 (57.1)	454 (40.8)
1	52,316 (9.4)	59 (5.3)
2	168,987 (30.2)	511 (45.9)
≥ 3	18,772 (3.4)	89 (8.0)
TPU level social factors		
Trust	2.44 ± 0.24	2.44 ± 0.24
Reciprocity norms	531.3 ± 202.6	540.1 ± 228.0
Moral norms	31.0 ± 10.0	31.6 ± 10.7
Number of religious organizations per capita	1.28 ± 0.58	1.47 ± 0.78
Number of nonprofit organizations per 10,000 people	0.30 ± 0.35	0.32 ± 0.40
Comorbidities		
Diabetes mellitus, n (%)	50,387 (9.0)	279 (25.1)
Cardiovascular disease, n (%)	5,698 (1.0)	51 (4.6)
Cerebrovascular disease, n (%)	2,326 (0.4)	19 (1.7)
COPD, n (%)	47,451 (8.5)	72 (6.5)
Asthma, n (%)	13,172 (2.4)	14 (1.3)
Hypertension, n (%)	99,094 (17.7)	552 (49.6)
Chronic kidney disease, n (%)	17,679 (3.2)	195 (17.5)
Charlson comorbidity index, n (%)		
0	239,523 (42.9)	153 (13.7)
1	175,191 (31.3)	229 (20.6)
≥ 2	144,355 (25.8)	731 (65.7)
Severe illness from COVID-19, n (%)	23,813 (4.3)	296 (26.6)
COVID-19-related death, n (%)	9,381 (1.7)	123 (11.1)

VI, visual impairment; SD, standard deviation; TPU, tertiary planning unit; COPD, chronic obstructive pulmonary disease.

Online supplemental Table S4. Moderation effect of the Delta strain on the association between severe visual impairment and secondary outcomes

Outcome	Exposure	Strain	OR (95% CI)	*P* for interaction
Severe illness	No VI	Low Delta period	1 (ref)	0.017
	Severe VI	Low Delta period	2.82 (2.07-3.84)	
	No VI	High Delta period	0.85 (0.82-0.88)	
	Severe VI	High Delta period	4.34 (3.61-5.23)	
COVID-19 related Death	No VI	Low Delta period	1 (ref)	0.934
	Severe VI	Low Delta period	2.11 (1.42-3.12)	
	No VI	High Delta period	1.61 (1.53-1.70)	
	Severe VI	High Delta period	3.50 (1.82-6.76)	

OR, odds ratio; CI, confidence interval; VI, visual impairment.

Online supplemental Table S5. Association of severe visual impairment with COVID-19 clinical outcomes in the entire cohort

Outcomes	Adjusted Odds Ratio (95% CI)	Events in Exposure Group (%)	Events in Non-Exposure Group (%)
Severe illness from COVID-19	2.25 (1.99-2.54)	372/3,799 (9.8)	5,367/111,998 (4.8)
Low prevalent period of the Delta strain	1.45 (1.22-1.72)	214/3,799 (5.6)	1,739/111,998 (1.6)
High prevalent period of the Delta strain	4.03 (3.41-4.77)	158/3,799 (4.2)	3,628/111,998 (3.2)
COVID-19-related death	1.25 (1.03-1.52)	121/3,799 (3.2)	2,679/111,998 (2.4)
Low prevalent period of the Delta strain	1.03 (0.73-1.47)	86/3,799 (2.3)	1,750/111,998 (1.6)
High prevalent period of the Delta strain	1.37 (1.09-1.71)	35/3,799 (0.9)	929/111,998 (0.8)

CI, confidence interval.

Online supplemental Table S6. Odds ratios and 95% CIs for different case-control ratios (1:k) in propensity score matching

Outcome	Total population				SARS-CoV-2 positive			
	**k = 1**	**k = 3**	**k = 5**	**k = 10**	**k = 1**	**k = 3**	**k = 5**	**k = 10**
SARS-CoV-2 infection	0.98 (0.85-1.12)	1.08 (0.96-1.20)	1.10 (0.99-1.22)	1.09 (0.99-1.20)				
Low Delta period	0.70 (0.56-0.87)	0.81 (0.68-0.97)	0.86 (0.73-1.02)	0.88 (0.75-1.04)				
High Delta period	1.09 (0.94-1.27)	1.17 (1.04-1.32)	1.19 (1.06-1.32)	1.17 (1.05-1.30)				
Severe illness	2.36 (1.93-2.90)	2.43 (2.09-2.83)	2.38 (2.07-2.74)	2.32 (2.03-2.63)	2.67 (2.90-3.42)	2.84 (2.34-3.44)	2.82 (2.36-3.37)	4.12 (3.47-4.88)
Low Delta period	1.43 (1.11-1.85)	1.50 (1.22-1.84)	1.53 (1.27-1.85)	1.52 (1.27-1.82)	1.61 (1.05-2.47)	1.82 (1.31-2.52)	1.80 (1.32-2.43)	2.65 (1.98-3.55)
High Delta period	4.81 (3.43-6.73)	4.77 (3.78-6.03)	4.25 (3.47-5.20)	4.04 (3.37-4.85)	3.25 (2.41-4.37)	3.43 (2.74-4.29)	3.39 (2.76-4.16)	4.85 (4.00-5.88)
Death	1.25 (0.94-1.67)	1.09 (0.87-1.36)	1.13 (0.91-1.39)	1.17 (0.95-1.43)	1.25 (0.92-1.68)	1.28 (1.01-1.62)	1.32 (1.05-1.65)	2.27 (1.82-2.84)
Low Delta period	0.99 (0.60-1.64)	0.91 (0.61-1.35)	0.97 (0.66-1.41)	1.05 (0.73-1.52)	1.85 (1.02-3.36)	1.28 (0.84-1.97)	1.31 (0.87-1.95)	2.13 (1.44-3.14)
High Delta period	1.41 (0.99-1.00)	1.19 (0.91-1.54)	1.20 (0.94-1.54)	1.22 (0.97-1.55)	1.14 (0.82-1.59)	1.28 (0.98-1.69)	1.33 (1.02-1.72)	2.34 (1.82-3.02)

CI, confidence interval.
